# Assessment of Serum Phosphate Levels in Chronic Kidney Disease Patients Across Different Stages of Kidney Disease

**DOI:** 10.7759/cureus.83051

**Published:** 2025-04-26

**Authors:** Arbab Muhammad Ali, Moazzam Ismail, Waqar Ahmad, Naveed Liaqat Ali, Khushhal Khan Nungyaal, Muhammad Sharif, Hayat Ullah, Muhammad Ayaz

**Affiliations:** 1 Department of Nephrology, Lady Reading Hospital Medical Teaching Institute (MTI) Peshawar, Peshawar, PAK; 2 Department of Internal Medicine, Mardan Medical Complex, Mardan, PAK; 3 Department of Health, District Headquarter Hospital, Mardan, PAK; 4 Department of Pathology, Mardan Medical Complex, Mardan, PAK; 5 Department of Pulmonology, Lady Reading Hospital Medical Teaching Institute (MTI) Peshawar, Peshawar, PAK

**Keywords:** chronic kidney disease, ckd complications, ckd stages, hemodialysis, hyperphosphatemia

## Abstract

Objective: This study aims to determine the serum phosphate level in patients diagnosed with chronic kidney disease (CKD) across different stages of the disease in a tertiary care hospital in Peshawar.

Materials and methods: This cross-sectional study was conducted in the Nephrology Department at Lady Reading Hospital, Peshawar, from August 2024 to January 2025. Over a six-month period, participants were recruited using a non-probability convenience sampling technique. The sample size of 296 was calculated using the WHO sample size calculator, with a 5% margin of error. The study included patients aged 18 years and older, of both sexes, diagnosed with any stage of CKD. Patients with acute kidney injury or significant disorders affecting phosphorus metabolism (e.g., primary hyperparathyroidism or malignancies) were excluded.

Results: A total of 296 patients with CKD from various etiologies were included in this study, consisting of 124 males (41.9%) and 172 females (58.1%). The age distribution was as follows: 20-39 years (17.6%), 40-49 years (26.7%), 50-59 years (29.4%), and ≥60 years (26.4%), with a mean age of 50.26 ± 12.14 years. Among the participants, 12.8% had CKD stage 2, 23% had stage 3, 33.4% had stage 4, and 30.7% had stage 5. Of the 296 patients, 77 (26%) were receiving hemodialysis, while 219 (74%) were on conservative management. The overall incidence of hyperphosphatemia was 56% (166 participants). Stage-wise, hyperphosphatemia was observed in 31.5% of CKD stage 2, 38.2% of stage 3, 72.7% of stage 4, and 61.5% of stage 5 patients.

Conclusion: The study has shown a higher burden of hyperphosphatemia among CKD patients, with an overall incidence of 56%. The prevalence of hyperphosphatemia increased with advancing CKD stages, peaking at CKD stage 4 (72.7%) and continuously remaining high in stage 5 (61.5%). These results stressed the need for early detection and effective management of hyperphosphatemia, particularly in the later stages of CKD, to lessen its associated complications. Moreover, the demographic distribution, with a mean age of 50.26 ± 12.14 years and a female predominance (58.1%), reflects the need for targeted interventions tailored to this patient population.

## Introduction

Chronic kidney disease (CKD) is a major health concern worldwide, affecting approximately 10-15% of the adult population [1,2]. It is distinguished by a gradual decline in renal function, which causes irregularities in mineral metabolism, including abnormal serum phosphate levels [3]. Hyperphosphatemia is a well-known CKD consequence that has been associated with poor clinical outcomes such as vascular calcification, cardiovascular disease, and an increase in mortality [4,5]. Understanding the variations in blood phosphate levels across CKD stages is critical for optimizing therapy regimens and improving patient outcomes [6].

The kidneys play an important role in maintaining phosphate homeostasis by controlling excretion and reabsorption [7]. As CKD advances, phosphate excretion decreases, resulting in a rise in blood phosphate levels, which promotes fibroblast growth factor-23 (FGF-23) production, ultimately lowering calcitriol synthesis [8]. This disturbance in phosphate balance is linked to secondary hyperparathyroidism (SHPT), bone mineral problems, and cardiovascular consequences [9]. However, phosphate anomalies can be seen in the early stages of CKD, making it necessary to measure phosphate levels continuously throughout the progression of the disease [10].

Previous research has demonstrated a strong association between phosphate dysregulation and negative clinical outcomes in CKD, underscoring the importance of timely and effective phosphate management [11-13]. While elevated phosphate levels are commonly observed, some CKD patients may present with normal or even low serum phosphate concentrations. These variations can be influenced by factors such as dietary phosphate intake, use of phosphate binders, and residual renal function [14]. Current guidelines emphasize the significance of monitoring and regulating blood phosphate levels in CKD patients, particularly in the late stages [15]. Phosphate-lowering therapy, such as dietary phosphate restriction, phosphate binders, and dialysis, is widely utilized to treat hyperphosphatemia and related complications [16]. However, phosphate levels vary across CKD stages, necessitating tailored treatment approaches to balance both the benefits and potential hazards of phosphate-lowering therapies [17].

The purpose of this study is to investigate serum phosphate levels in CKD patients at various stages of the disease, offering insight into their patterns, contributory variables, and potential implications for clinical management. By detecting phosphate abnormalities at various stages, this study will help us better understand phosphate homeostasis in our local CKD populations and aid in the development of targeted approaches to treatment [18,19].

## Materials and methods

This cross-sectional study was conducted in the Nephrology Unit at Lady Reading Hospital (MTI), Peshawar, from August 2024 to January 2025 after obtaining approval from the Institutional Review Board at Lady Reading Hospital. A non-probability convenience sampling technique was used. The sample size was calculated using the WHO sample size calculator, assuming a 74% prevalence of hyperphosphatemia in CKD patients, a 95% confidence level, and a 5% margin of error, resulting in a required sample size of 296 participants [20].

Adults aged 18 years and older residing in Peshawar, Pakistan, with CKD stages 2 to 5 or on dialysis, who provided written informed consent, were included. CKD staging was based on estimated glomerular filtration rate (eGFR) calculated using the Modification of Diet in Renal Disease (MDRD) equation. CKD stage 2 was defined as an eGFR of 60-89 mL/minute/1.73 m², stage 3 as 30-59, stage 4 as 15-29, and stage 5 as <15 mL/minute/1.73 m² or dialysis-dependent [21].

The exclusion criteria included patients with acute kidney injury (as evidenced by a recent rise in serum creatinine within the past seven days), acute infections, active inflammatory diseases, uncontrolled thyroid dysfunction, or conditions directly affecting phosphate metabolism, such as primary hyperparathyroidism, vitamin D toxicity, tumor lysis syndrome, or malignancies involving bone metastases.

Eligible patients were recruited from the Nephrology Outpatient Department (OPD) of Lady Reading Hospital. Known CKD patients visiting the OPD for routine follow-up or laboratory testing were approached. As serum phosphate is not always routinely assessed in all stages of CKD, a repeat serum phosphate test was explicitly requested for the purpose of the study, following verbal explanation and written informed consent. A 5 mL blood sample was drawn from each participant and transported immediately to the hospital laboratory for phosphate analysis, which was conducted under the supervision of a certified pathologist.

Additional data such as age, gender, weight (kg), height (cm), body mass index (BMI), duration of CKD, and comorbidities like diabetes mellitus and hypertension were recorded using a structured proforma. Strict adherence to the inclusion and exclusion criteria helped reduce selection bias.

Data were analyzed using IBM SPSS Statistics for Windows, Version 24 (Released 2016; IBM Corp., Armonk, New York). Quantitative variables (e.g., serum phosphate, creatinine, eGFR, and BMI) were presented as means and standard deviations. Categorical variables (e.g., gender, CKD stage, presence of hyperphosphatemia, hypertension, and diabetes) were summarized as frequencies and percentages. Data were stratified by age group, gender, and CKD stage. Associations between categorical variables were assessed using chi-square tests. A p-value ≤ 0.05 was considered statistically significant. The results were presented in tables and graphs for clarity and interpretation.

## Results

A total of 296 patients with CKD of various causes were enrolled in this research, consisting of 172 (58.1%) females and 124 (41.9%) males. The mean age of the participants was 50.26 ± 12.14 years, with an age distribution as follows: 52 (17.6%) patients were aged 20-39 years, 79 (26.7%) were 40-49 years, 87 (29.4%) were 50-59 years, and 78 (26.4%) were ≥60 years. The mean age for females was 49.71 years, while it was 49.14 years for males.

Regarding CKD staging, 38 (12.8%) had CKD stage 2, 68 (23%) had stage 3, 99 (33.4%) had stage 4, and 91 (30.7%) had CKD stage 5. The mean serum phosphate level in dialysis-dependent stage 5 patients was 5.71 mg/dL, while it was 4.89 mg/dL among non-dialysis stage 5 patients.

Among the enrolled patients, 77 (26%) received hemodialysis, while 219 (74%) were treated conservatively. The mean serum phosphate level for dialysis-dependent patients was 5.71 mg/dL, while for non-dialysis-dependent patients, it was 4.44 mg/dL. The normal range for serum phosphate levels in adults is generally 2.5 to 4.5 mg/dL, and both patient groups had elevated phosphate levels compared to this reference range.

**Figure 1:**
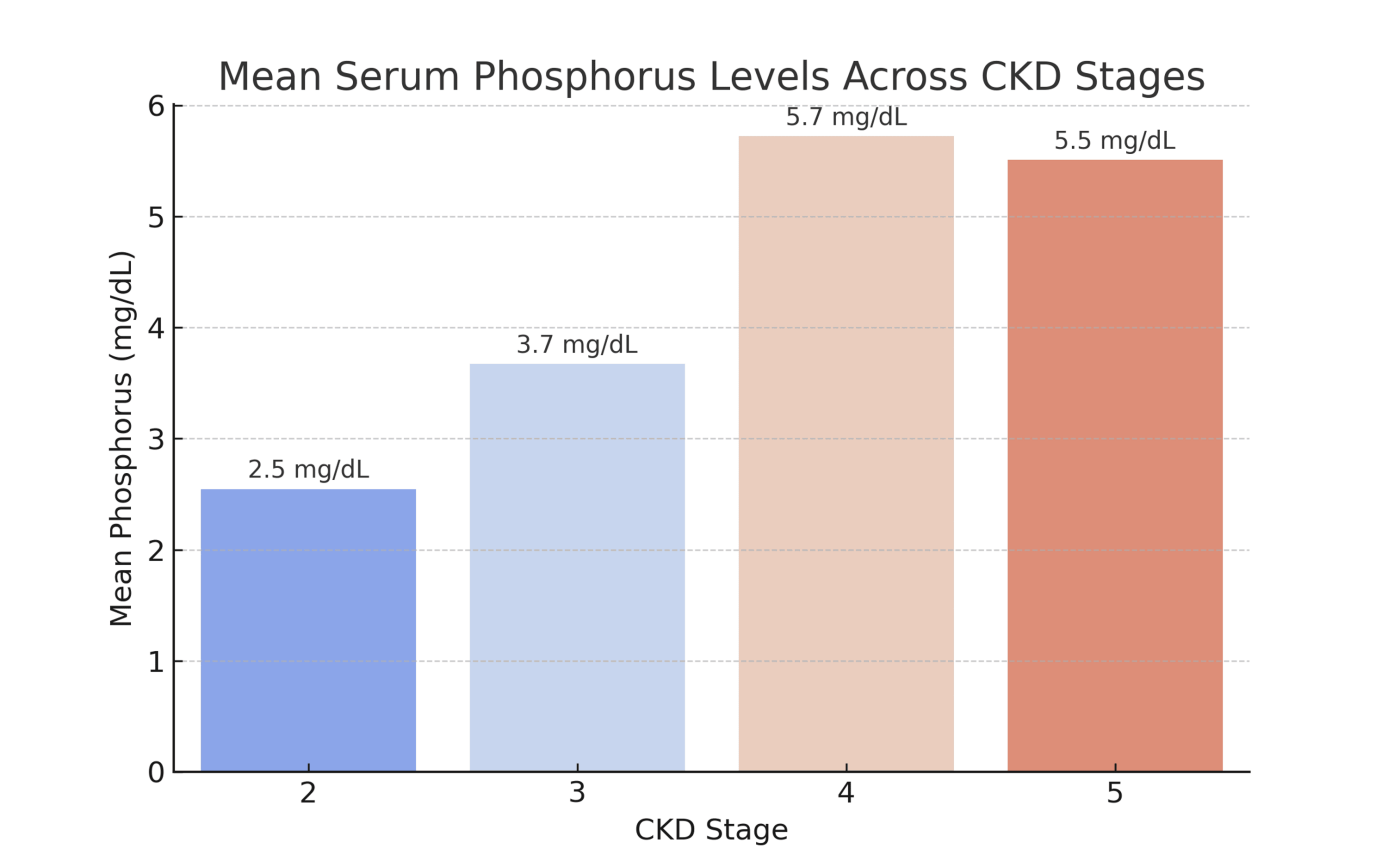
Mean serum phosphorus levels across various stages of CKD CKD: chronic kidney disease

**Figure 2:**
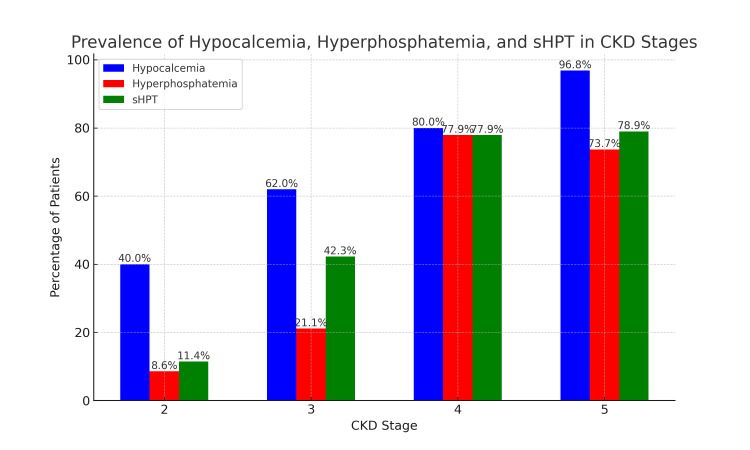
Prevalence of hypocalcemia, hyperphosphatemia, and secondary hyperparathyroidism among the participants sHPT: secondary hyperparathyroidism; CKD: chronic kidney disease

In our study, 166 participants (56%) had an overall incidence of hyperphosphatemia. The mean serum phosphorus levels showed an increasing trend across CKD stages (Figure 1). Table 1 displays the stage-wise frequency of hyperphosphatemia. An ANOVA test revealed a highly significant difference (p < 0.0001) among the stages, indicating a progressive rise in serum phosphorus levels with advancing CKD. Figure 2 illustrates the incidence of hypocalcemia, hyperphosphatemia, and SHPT among the participants.

**Table 1:** Frequency of hyperphosphatemia in various CKD stages CKD: chronic kidney disease

CKD Stage	Hyperphosphatemia (Yes)	Hyperphosphatemia (No)	Total
Stage 2	12 (31.6%)	26 (68.4%)	38 (100%)
Stage 3	26 (38.2%)	42 (61.8%)	68 (100%)
Stage 4	72 (72.7%)	27 (27.3%)	99 (100%)
Stage 5	56 (61.5%)	35 (38.5%)	91 (100%)
Total	166 (56.1%)	130 (43.9%)	296 (100%)

## Discussion

This study assessed serum phosphate levels across different stages of CKD in a tertiary care setting in Peshawar. Our findings demonstrate a progressive rise in serum phosphate levels with worsening renal function, a trend consistent with existing literature. A study of Japanese non-dialysis-dependent CKD patients found that higher serum phosphorus levels (≥3.7 mg/dL) were associated with an increased risk of CKD progression [22]. Longitudinal analysis of CKD patients progressing to end-stage kidney disease (ESKD) showed that serum phosphate levels increased modestly over time, with changes becoming evident approximately five years before ESKD onset [23]. Similarly, in our study, the mean serum phosphorus levels increased significantly across CKD stages, with peak levels observed in advanced stages (CKD stages 4 and 5), establishing a strong connection with a previous study conducted in Karachi, Pakistan [24].

In particular, 56% of participants had hyperphosphatemia, revealing the high burden of phosphate imbalance in CKD patients. This prevalence is notably higher than reported in some previous studies, which generally observe lower rates of hyperphosphatemia, especially in early stages or with phosphate management strategies [25,26]. However, our findings align with studies from institutions such as Mayo Hospital, Lahore, which have documented phosphate retention and SHPT as common complications in advanced CKD stages, especially when renal function deteriorates [27].

While hyperphosphatemia is often seen in CKD patients, SHPT, which is triggered by phosphate retention, may be a more accurate reflection of CKD-MBD (mineral and bone disorder). SHPT is strongly associated with declining renal function and impaired phosphate excretion [28]. This study supports the well-established relationship between declining GFR and phosphate retention. Our results demonstrate a statistically significant difference in serum phosphate levels across CKD stages (p < 0.0001), emphasizing the impact of kidney dysfunction on phosphate metabolism.

Interestingly, while a statistically significant difference was seen in the mean age between males and females (p < 0.05), serum phosphate levels did not differ significantly between genders (p = 0.348). This finding contrasts with some reports suggesting gender-based variations in phosphate metabolism, which may be influenced by hormonal differences and dietary intake. Further investigation with a larger sample size could clarify whether gender-related factors play a role in phosphate homeostasis among CKD patients in our region.

The clinical inference of our study findings is remarkable, reinforcing the need for early phosphate monitoring and dietary changes in CKD patients, specifically in those progressing to advanced stages. The mean serum phosphate level for hemodialysis-dependent patients was 5.71 mg/dL, while for non-hemodialysis-dependent patients, it was 4.44 mg/dL. However, this difference suggests a lack of a significant impact of dialysis on phosphate levels in our cohort, challenging the typical expectation that dialysis would lead to better phosphate control. These findings are in accordance with reports from a previous study in India, which indicated that serum phosphate levels rise significantly in advanced CKD stages, particularly in dialysis-dependent patients, where mean levels can reach 6.12 mg/dL compared to 4.56 mg/dL in non-dialysis patients [29]. Since 26% of patients were on hemodialysis while 74% were managed conservatively, the findings suggest that phosphate control strategies should be tailored based on disease severity and treatment modality.

This study has certain limitations. First, it is a single-center research, which may restrict the findings' applicability to larger CKD populations. Second, the research design is cross-sectional, which precludes assessing long-term patterns in blood phosphate levels and their influence on disease development. Third, we did not account for dietary phosphate consumption, medication adherence, or the use of phosphate binders, all of which might affect blood phosphate levels. Fourth, additional variables influencing phosphate homeostasis, such as FGF-23 levels and vitamin D status, were not studied, which might provide further information about phosphate metabolism in CKD. Finally, even with rigorous data collection, possible confounders such as comorbidities and dialysis adequacy may influence the outcomes. Future multicenter, longitudinal studies integrating these parameters might improve our understanding of hyperphosphatemia in CKD.

## Conclusions

This study highlights the progressive rise in serum phosphate levels with advancing CKD stages, indicating the growing burden of hyperphosphatemia in advanced kidney disease. Our study demonstrates that serum phosphate levels remain relatively stable in early CKD stages but increase significantly in CKD stages 4 and 5, indicating the declining renal phosphate excretion. This emphasizes the importance of early monitoring and management to avoid disorders associated with hyperphosphatemia, such as vascular calcification, SHPT, and cardiovascular disease.

The findings highlight the necessity of dietary phosphate restriction, phosphate binders, and optimum dialysis techniques for efficiently managing serum phosphate levels. Given the close link between hyperphosphatemia and CKD progression, early management can help prevent CKD-MBD and lower long-term morbidity. Future studies should look at long-term changes in phosphate management and the influence of new therapy alternatives. A proactive, interdisciplinary strategy is required to enhance phosphate homeostasis and improve clinical outcomes in CKD patients.
